# The Effect of Chewing Gum on Anxiety and Labor Pain: A Systematic Review and Meta‐Analysis

**DOI:** 10.1002/hsr2.71447

**Published:** 2025-11-10

**Authors:** Farideh Mohsenzadeh‐Ledari, Hoda Shirafkan, Mina Galeshi

**Affiliations:** ^1^ Nursing Care Research Center, Health Research Institute Babol University of Medical Sciences Babol Iran; ^2^ Social Determinants of Health Research Center, Health Research Institute Babol University of Medical Sciences Babol Iran; ^3^ Clinical Research Development Unit of Rouhani Hospital Babol University of Medical Sciences Babol Iran

**Keywords:** anxiety, chewing gum, labor pain

## Abstract

**Background and Aims:**

Childbirth as a physiological phenomenon is painful. Fear and anxiety about labor pain increase the likelihood of women choosing cesarean delivery. Nonpharmacological interventions may enhance maternal comfort during labor. This study investigated the effect of chewing gum on anxiety and labor pain.

**Methods:**

Studies from 2000 to 2024 were identified through searches of Web of Science, PubMed, Scopus, Google Scholar, and Embase, with no restrictions on language or geographical location. Meta‐analysis using Stata v17 employed a random‐effects model to calculate standardized mean differences (SMD) with 95% confidence intervals (CIs). Heterogeneity was evaluated using the *I* ^2^ statistic.

**Results:**

An analysis of five trials (326 women, high to low quality, varying inclusion criteria) showed that chewing gum for 20 min in active and second phases significantly reduced pain and anxiety. Pain scores were lower in the gum‐chewing group during the active (SMD: −1.23, 95% CI: −2.30 to −0.16, *p* < 0.00, *I* ^2^ = 93.97%) and transition phases (SMD: −1.63, 95% CI: −2.35 to −0.90, *p* = 0.00, *I* ^2^ = 85.17%) and anxiety scores were also reduced (SMD: −1.44, 95% CI: −1.03 to 3.92, *p* < 0.00, *I* ^2^ = 98.41% and SMD: −0.55, 95% CI: −0.79 to −0.30, *p* = 0.00, *I* ^2^ = 0.00%, respectively).

**Conclusion:**

Chewing gum use may reduce labor pain and anxiety and shorten labor duration, according to this study. However, more rigorous research is necessary to confirm these findings.

## 1 Introduction

Childbirth is often portrayed as a painful yet miraculous part of a woman's life [1]. Pain is an unpleasant physiological experience that medical science has long sought to manage and alleviate [2]. Fear of childbirth often leads women to prefer cesarean delivery over natural vaginal birth [3]. Severe labor pain is experienced by approximately 60% of first‐time mothers and 40% of mothers who have given birth previously [4, 5].

Severe maternal pain during labor can negatively impact the newborn, potentially causing delayed heart rate recovery, lower Apgar scores, compromised fetal heart sounds, uteroplacental insufficiency, and fetal acidosis. Pain management is therefore crucial for positive labor outcomes [6].

Maternal anxiety during labor can influence labor progression, leading to prolonged bleeding, heightened pain, negative labor experiences, psychological distress, postpartum depression, and delayed labor onset. Maternal psychological stress correlates with labor pain and anxiety, highlighting the importance of pain management [7]. Maternal mental stress can intensify childbirth pain and anxiety. Addressing these factors is crucial to mitigating debilitating labor pain. Employing nonmedical strategies can significantly enhance nursing care to alleviate labor pain and anxiety. Techniques like mindfulness, breathing exercises, and a supportive presence during labor can greatly reduce the mother's distress. These methods encourage physical relaxation and empower mothers by giving them a sense of control over their birthing experience. Additionally, having a doula or supportive partner can further improve the environment by providing emotional reassurance and practical assistance throughout labor [8, 9].

Distraction techniques are a common, nonpharmacological approach to reduce pain and discomfort, particularly during medical procedures. These techniques compete with the pain pathway, and the cognitive process is similar to that of peripheral pain modulation, leading to a decreased sensation and perception of pain. Chewing gum is a subset of these distraction methods. Chewing gum can alleviate stress and anxiety by providing a distraction, as demonstrated in studies of both chronic and acute stress [10, 11].

Studies indicate that the most effective strategies for alleviating pain and anxiety are those that are easily accessible, simple to implement, require no prior training, and are controlled by the individual. Among nonpharmacological methods, chewing gum is particularly notable due to its affordability, ease of use, and noninvasive nature. It also enhances the mother's sense of confidence and active participation during the process [12].

Given the lack of systematic reviews and meta‐analyses on reducing elective Cesarean section rates and finding viable strategies for labor pain reduction, this study aims to determine if chewing gum, as a nondrug alternative, affects anxiety levels, labor stage durations, labor pain, and clinical complications in individuals.

## 2 Methods

This study adhered to PRISMA guidelines, was approved by the Institutional Review Board at Babol University of Medical Sciences (IR.MUBABOL.HRI.REC.1402.247), and registered with PROSPERO (CRD42024528967) on April 6, 2024.

### 2.1 Eligibility Criteria

This study included all randomized controlled trials comparing chewing gum use during labor with a control group. Studies combining chewing gum with other interventions were excluded. Outcome measures included VAS scores and the Spielberger State‐Trait Anxiety Inventory (STAI).

### 2.2 Search Strategies

We searched Web of Science, PubMed/MEDLINE, Scopus, Google Scholar, and Embase using database‐specific syntax. A manual search of references from included studies, key journals, conference/congress research papers, and theses was also performed. The search, conducted from 2000 to 2024 without language or geographical limitations, employed MeSH, Entree, and free text methods. The primary search terms were “chewing gum,” “labor pain,” and “Anxiety.”

### 2.3 Study Selection

Three independent reviewers searched the databases. Study selection involved initial title/abstract screening, followed by full‐text screening of eligible studies, with duplicates removed. A third reviewer resolved disagreements.

### 2.4 Quality Assessment

D.K. and A.C. independently assessed the risk of bias (RoB) in RCTs using the Cochrane Collaboration ROB‐2 tool. For individually randomized trials, RoB was evaluated across five domains [1]: randomization process [2]; deviations from intended interventions [3]; missing outcome data [4]; measurement of the outcome; and [5] selection of reported results. Domain 1 was assessed at the study level, while the remaining domains were assessed at the outcome level. Cluster‐RCTs were assessed similarly, but with two domains addressing allocation bias instead of domain 1: [1a] the randomization process and [1b] the identification or recruitment of participants [13]. Each domain was judged to have a high, low, or unclear RoB. Discrepancies were resolved through discussion, re‐evaluation of evidence, and consultation of guidelines, potentially involving a third reviewer for independent assessment. This systematic approach ensures transparent and fair RoB decisions, improving assessment reliability.

### 2.5 Data Mining

Two independent reviewers extracted data from included studies using a standardized form. Extracted details included: first author, publication year, sample size (cases/controls), and intervention.

### 2.6 Data Analysis

Meta‐analysis (Stata 17.0) used the standardized mean difference (SMD) for the VAS score. Heterogeneity was evaluated using *I* ^2^ (0%−25%: very low, > 75%: high) and the chi‐square test. A random‐effects model calculated SMD and 95% confidence intervals (CIs). Network meta‐analysis and diagrams were generated in Stata v17.0. Inconsistency was assessed with a *Z*‐test comparing direct and indirect results (*p* > 0.05 indicated no inconsistency). Publication bias was assessed using funnel plots, Egger's test, and Begg's test (*p* > 0.10 indicated no bias; *p* < 0.10 indicated significant bias).

## 3 Results

### 3.1 Characteristics of the Included Studies

**Figure 1:**
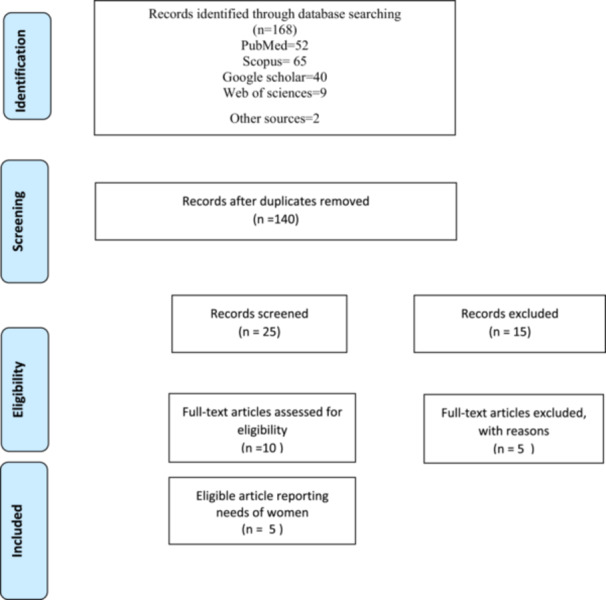
Flow diagram of study selection.

The first search yielded 168 articles, which were reduced to 140 after removing duplicates. Title and abstract review excluded 130 articles, leaving 10 for the full‐text review. Five articles were excluded during the full text review, and one additional article was identified through citation searching (Figure 1).

**Figure 2:**
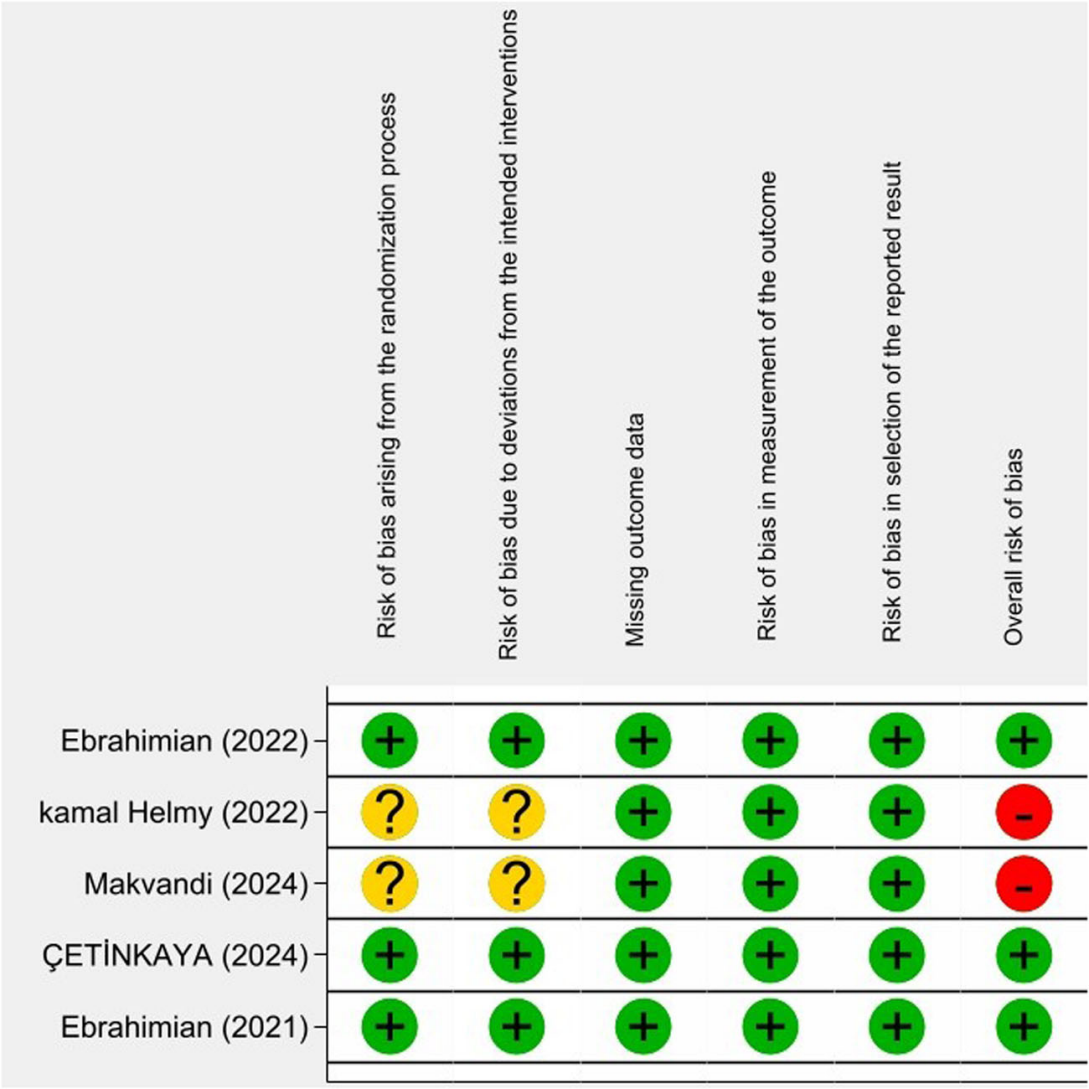
Traffic light plot.

**Figure 3:**
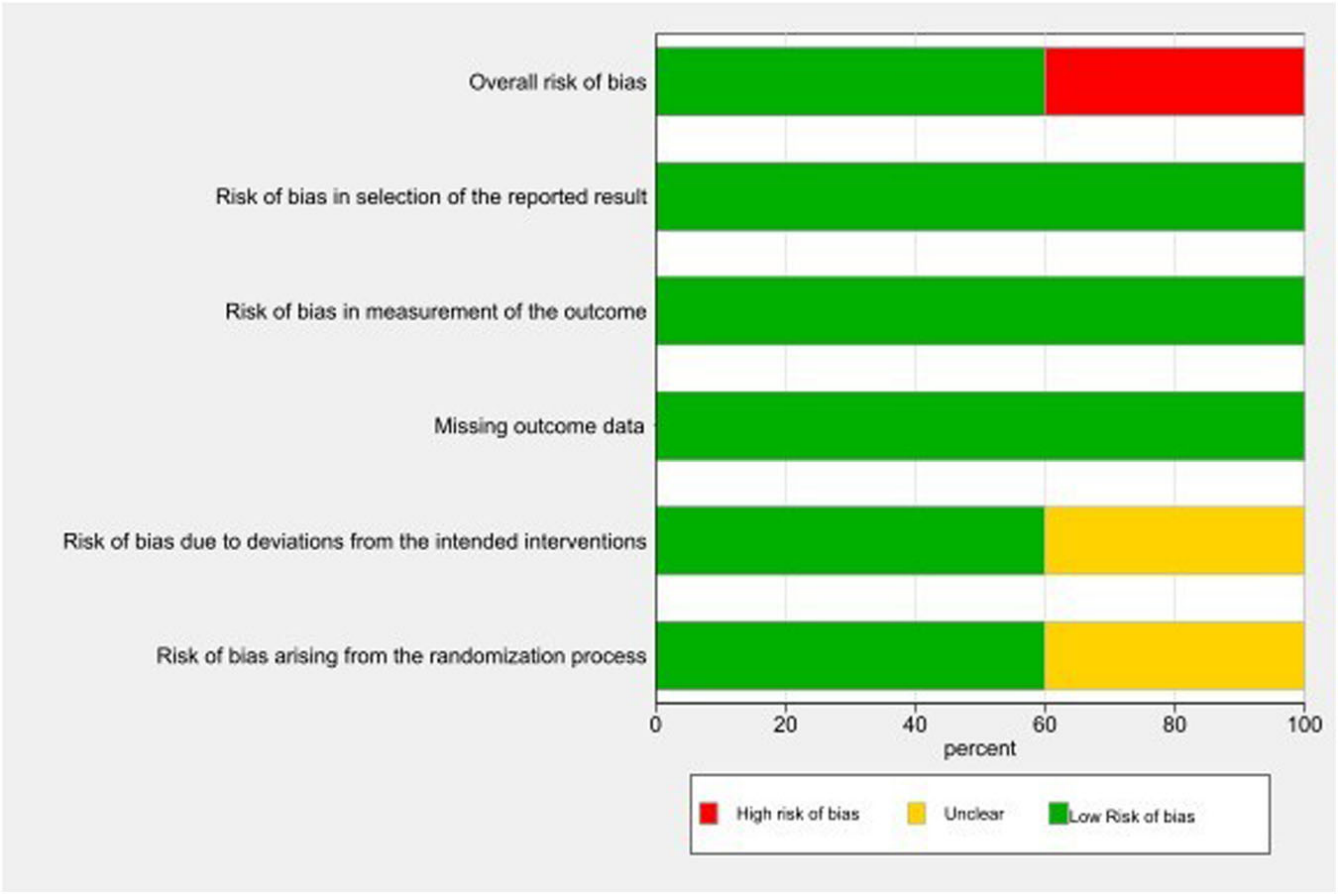
Summary plot.

No quasi‐randomized trials were included. Distribution bias, publication bias, assessed with Begg's and Egger's tests, was not significant (*p *= 0.70 and 0.82, respectively). The included trials were generally low quality, with most exhibiting high or unclear RoB across most Cochrane domains (Figures 2 and 3). Three studies had a low RoB, two had an unclear risk, and the majority had a high RoB. Table 1 presents the characteristics of the included clinical trials. All the studies used chewing gum as an intervention for labor pain and anxiety. The included studies were distributed across the following countries: Iran (three studies), Egypt (one study), and Turkey (one study). In most of the included trials, chewing gum was used during the active phase and transition phase. Most of the studies described in points of interest how the results were evaluated.

**Table 1:** The main characteristics of the included studies.

No	Author (year)	Study location	Sample size	Outcome	Intervention
1	Şi̇mşek Çeti̇nkaya and (2024) [14]	Turkey	64	Pain; duration of delivery; birth satisfaction	Chewing gum interventions in 3 cm dilatations for 20 min
2	Ebrahimian et al. 2022 [2]	Iran	93	Pain; anxiety	Chewing gum interventions twice in 4–5 cm and 7–8 cm dilatations for 20 min
3	kamal Helmy et al. 2022 [3]	Egypt	300	Pain; anxiety; patient satisfaction	Chewing gum interventions performed twice in 4–5 cm and 7–8 cm dilatations for 20 min
4	Ebrahimian et al. 2021 [15]	Iran	93	Duration of delivery; birth satisfaction	Interventions performed twice: in the active (4–5 cm) and second (7–8 cm) phases of parturition for 20 min
5	Makvandi et al. 2013 [4]	Iran	66	Pain; anxiety; labor stages duration	Chewing gum interventions for 30 min in 3–5 cm dilatation

### 3.2 Synthesis of Results: Effects of Interventions

No quasi‐randomized trials were included; distribution bias was insignificant (Begg's test, *p* = 0.70; Egger's test, *p* = 0.82). The included trials were generally of low quality, with most exhibiting a high or unclear RoB across most Cochrane RoB domains (Figures 2 and 3). Only three studies had a low RoB, while two had an unclear risk, and the remainder had a high risk.

Table 1 summarizes the characteristics of the included clinical trials. All studies used chewing gum as an intervention for labor pain and anxiety, and were conducted in Iran (three studies), Egypt (one study), and Turkey (one study). Chewing gum was typically administered during the active and transition phases of labor. Most studies adequately described how the results were evaluated.

### 3.3 Synthesis of Results: Effects of Interventions

#### 3.3.1 Labor Pain Score

**Figure 4:**
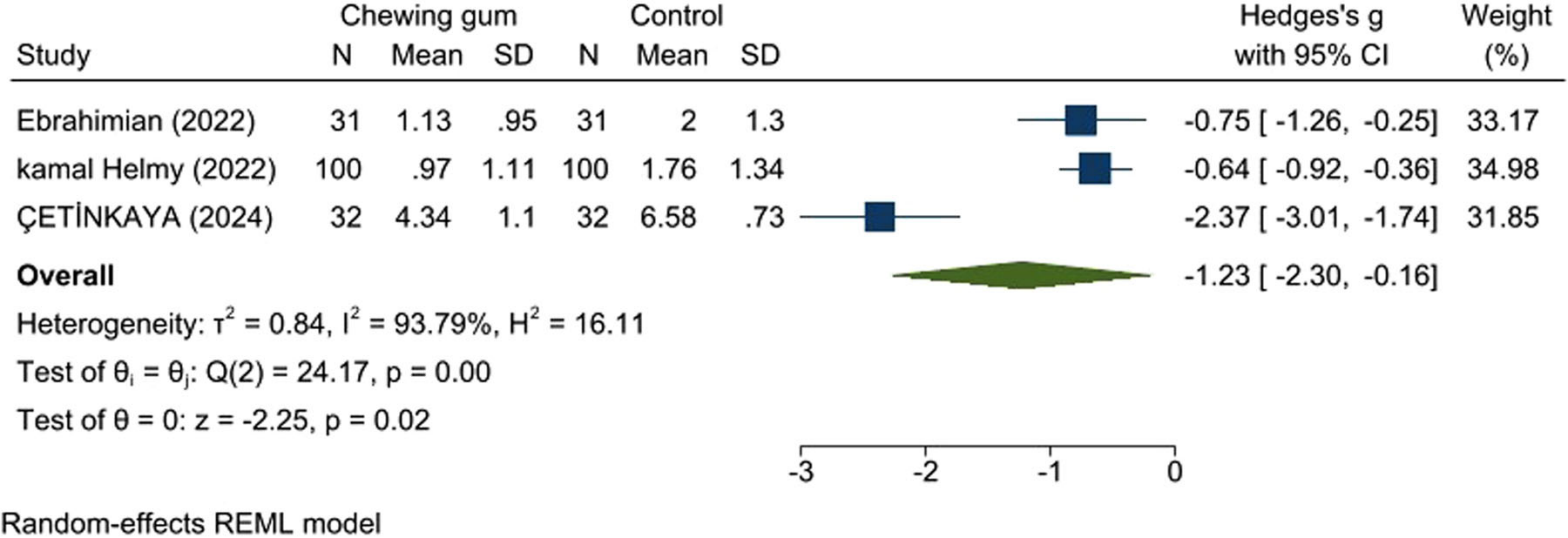
Pain score in the active phase.

**Figure 5:**
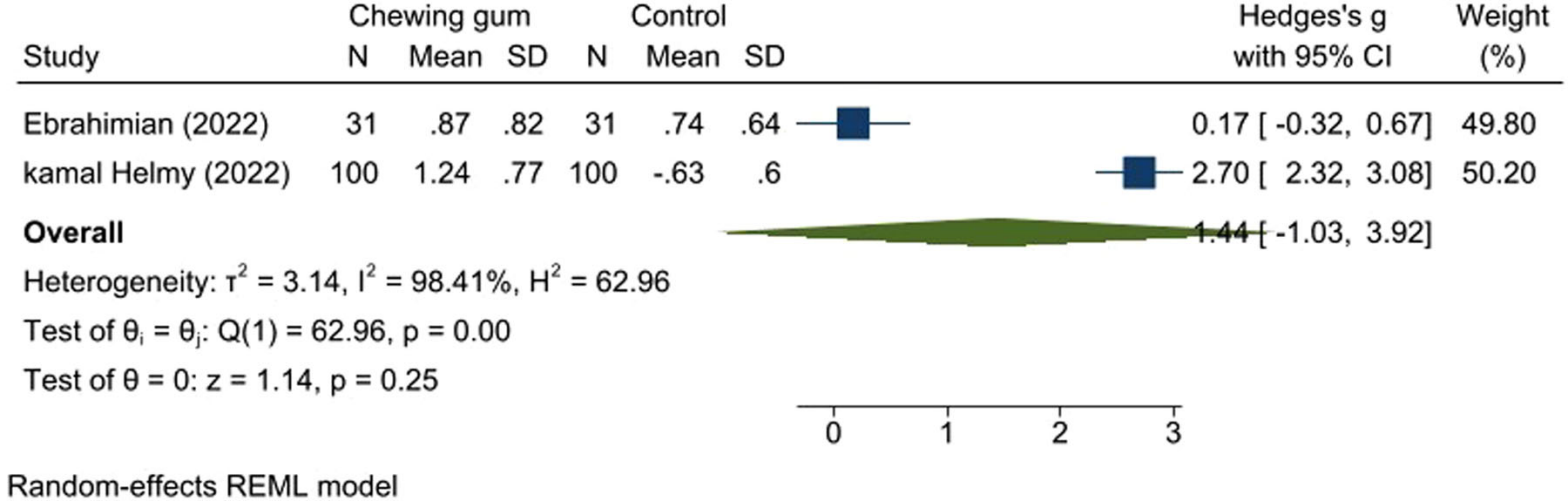
Pain score in the transitional phase.

A review of three studies (326 participants) indicated that gum chewing significantly reduced pain. One study had some concern for bias [14], while the others had a low RoB [16], [17]. Gum chewing reduced pain scores during the active and transition phases (SMD: −1.23, 95% CI: −2.30 to −0.16, *p* < 0.00, *I* ^2^ = 93.97% and SMD: −1.44, 95% CI: −1.03 to 3.92, *p* < 0.00, *I* ^2^ = 98.41%, respectively) (Figures 4 and 5).

#### 3.3.2 Active Phase and the Second Stage of Labor Duration

**Figure 6:**
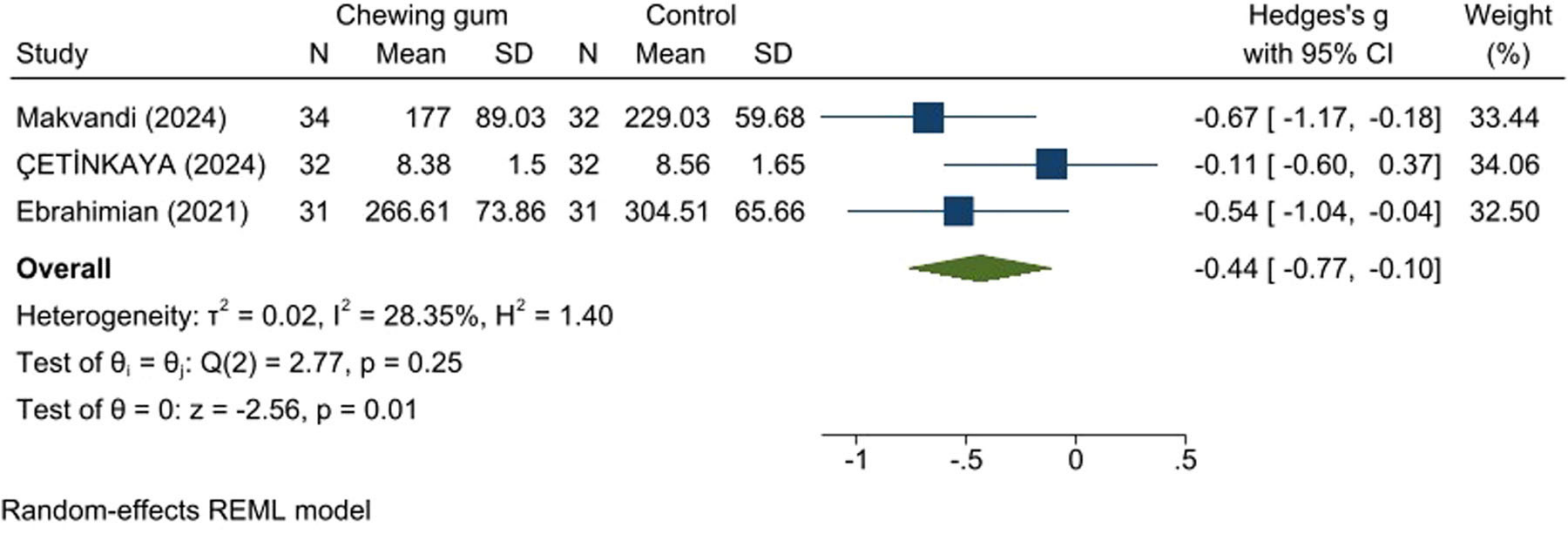
Active phase duration.

**Figure 7:**
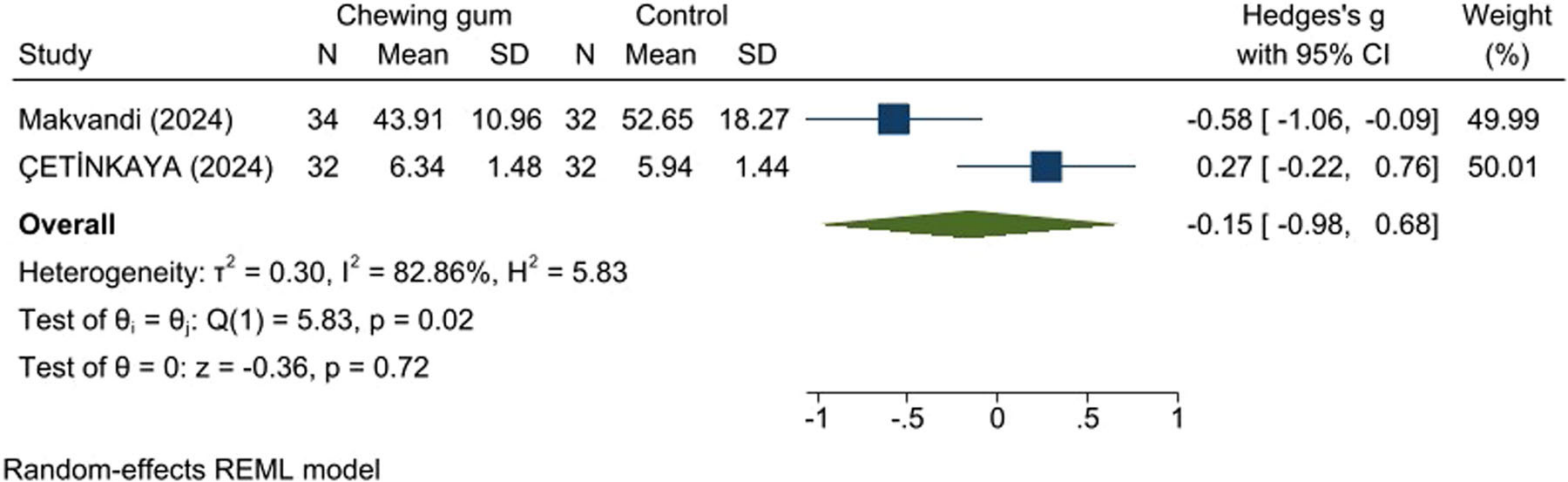
Second stage of labor duration.

Gum chewing was associated with a significantly shorter active phase (SMD: −0.44, 95% CI: −0.77 to −0.10, *p* = 0.01, *I* ^2^ = 28.35%) (Figure 6) and second stage of labor (SMD: −0.15, 95% CI: −0.98 to −0.67, *p* = 0.02, *I* ^2^ = 82.86%) (Figure 7) compared to the control group.

#### 3.3.3 Anxiety Score in Labor

**Figure 8:**
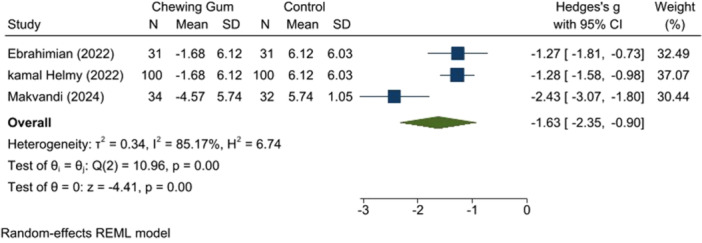
Anxiety score in active phase.

**Figure 9:**
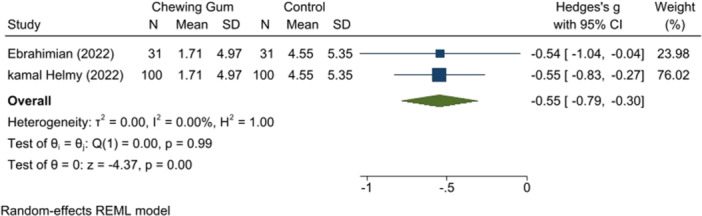
Anxiety score in transitional phase.

Two studies (*n* = 130) with low RoB [14, 18] indicated that chewing gum reduced anxiety levels during the active phase (SMD: −1.63, 95% CI: −2.35 to −0.90, *p* = 0.00, *I* ^2^ = 85.17%). The transition phase also showed a decrease in anxiety in the chewing gum group (SMD: −0.55, 95% CI: −0.79 to −0.30, *p* = 0.00, *I* ^2^ = 0.00%) (Ebrahimian and Kamal study) (Figures 8 and 9).

## 4 Discussion

This study suggests that gum chewing significantly reduces labor pain and anxiety. Childbirth can be an upsetting experience for pregnant individuals, who often experience anxiety about the birthing process. While this stress is largely psychological, it is significant and can cause unease. Akifusa et al.'s study suggests chewing gum effectively promotes relaxation [19].

Chewing influences salivary cortisol levels, a marker of stress [20]. Chewing gum may reduce anxiety and stress by balancing cortisol levels [21, 22]. Chewing may reduce stress by stimulating the release of mood‐regulating neurotransmitters like serotonin and dopamine. This rhythmic, mild exercise increases blood flow and relaxes jaw muscles, fostering a sense of well‐being [23]. Chewing gum can effectively reduce pain by diverting the brain's focus. It shifts attention away from rumination, lessens the neurological response to painful stimuli, and elevates mood. In some instances, it can even alleviate pain or discomfort. Compared to analgesics, chewing gum is a noninvasive, affordable, and convenient alternative with fewer side effects [24] Chewing gum also offers other benefits [25]. In this study, women within the chewing gum group experienced less anxiety. which is consistent with the study of Ebrahimian et al [16], and Baraia [17]. In light of these findings, healthcare professionals might study incorporating chewing gum into pain management strategies, especially for patients who prefer nonpharmacological options. As more studies emerge, the potential of chewing gum as a simple yet effective tool for enhancing well‐being and reducing pain will become increasingly evident. In conclusion, while it may seem trivial, the act of chewing gum holds promise as a valuable adjunct in pain management and mental health support, warranting further exploration and consideration in both clinical and everyday settings.

In the investigation conducted in this study, the length of the active phase and the second stage of labor was significantly less in the intervention group than in the control group which was consistent with the study of Makundi et al. [18], Ebrahimian et al. [15], and Şi̇mşek Çeti̇nkaya and Durmuş [14]. It seems that due to the reduction in the level of anxiety the reduction in the length of the stages of childbirth in the present study is; As Kaviani et al also observed during a study that by reducing women's anxiety by ice massage at the acupressure point, the length of the first and second stage of labor is significantly reduced in comparison with the control group [26]. Gaudernack et al. also concluded in their study that different types of anxiety during childbirth increase the length of the stages of childbirth [27].

Unlike more traditional methods such as breathing exercises, massage, and acupressure, which have been widely researched and recommended for their calming effects, chewing gum offers a unique approach that combines sensory engagement with potential psychosomatic benefits [28]. Breathing exercises typically focus on controlled inhalation and exhalation to foster relaxation and manage pain signals. This method requires conscious effort and mental focus, which can be challenging during the intense experience of labor [29]. On the other hand, chewing gum provides a more automatic, hands‐on distraction that may ease tension by keeping the mind engaged in a rhythmic, repetitive task. Chewing may boost brain blood flow and endorphins, easing pain and promoting relaxation [30, 31].

Massage and acupressure, while beneficial for their ability to relieve muscle tension and promote a sense of well‐being [25], often require the presence of a partner or healthcare provider. In contrast, chewing gum is an easily accessible and portable intervention that can be employed at any moment during labor, giving the birthing person a sense of agency and control over their experience. The independent nature of gum chewing allows for a personal coping mechanism that can be tailored to individual preferences without reliance on external support.

Breathing exercises and acupressure often require prior learning and practice to be effective, while chewing gum can be used instinctively during labor without any advanced training. This ease of use makes it an appealing choice for those who may feel overwhelmed or unprepared.

However, chewing gum's effectiveness in reducing labor pain and anxiety differs from person to person. Some may benefit from its sensory distraction, while others might find relief through the calming focus of deep breathing or the physical comfort provided by massage techniques. Incorporating chewing gum into a broader range of nonpharmacological pain management strategies could enhance the overall labor experience, serving as a simple and effective complement to other methods.

In conclusion, although chewing gum cannot replace established nonpharmacological interventions like breathing exercises, massage, or acupressure, it offers an additional layer of support that can be conveniently utilized during labor. With its unique combination of distraction, sensory engagement, and self‐empowerment, chewing gum is a valuable option among coping strategies for managing labor pain and anxiety.

## 5 Strengths and Limitations

This meta‐analysis has notable limitations. Variations in gum chewing interventions among the RCTs likely explain the heterogeneity. Furthermore, the included studies generally exhibit low quality, with many lacking reports on randomization concealment and blinding. While blinding participants is difficult, observers can be blinded to minimize bias. Future research should prioritize better RCT design, and with most research focused on Asian countries. This restricts the generalization of findings to other regions and raises concerns about cultural biases. The narrow scope of current literature limits the applicability of results and the development of comprehensive theories. Future research should involve scholars from underrepresented regions to achieve a broader understanding of these phenomena. Incorporating diverse perspectives will improve the validity of findings and enrich the discourse on human behavior across different societies. Our meta‐analysis is strengthened by a comprehensive search of five international databases, including gray literature, without language restrictions, covering studies up to December 2024.

## 6 Conclusion

The discoveries of the present study show that chewing gum can possibly decrease labor pain and anxiety in women. Chewing gum shows up to be a cost‐effective, promptly accessible, and well‐tolerated mediation in labor. Be that as it may, the tall heterogeneity watched among included studies shows potential inconsistency in understanding reactions to chewing gum and highlights the require for advance inquire about to get it variables impacting its viability. Too, future studies may center on standardizing gum chewing convention in labor to decide the ideal recurrence and term for most extreme benefits.

## Author Contributions

**Farideh Mohsenzadeh‐Ledari and Mina Galeshi:** conceptualization, writing – original draft, writing – review and editing, methodology, project administration, validation.

## Conflicts of Interest

The authors declare no conflicts of interest.

## Transparency Statement

The lead author, Mina Galeshi, affirms that this manuscript is an honest, accurate, and transparent account of the study being reported; that no important aspects of the study have been omitted; and that any discrepancies from the study as planned (and, if relevant, registered) have been explained.

## Data Availability

All relevant data are within the paper; however, any question or other file data is required. You can contact us using the email address upon reasonable request (galeshi_m@yahoo.com).
